# Discitis Mimicking Septic Arthritis in a 17-Month-Old Boy

**DOI:** 10.7759/cureus.67460

**Published:** 2024-08-22

**Authors:** Tariq Aziz, Muneebah Ihsan, Abdelfatah M Elsenosy, Aamir Saleem, Abdullah Zakaullah

**Affiliations:** 1 Emergency Medicine, University Hospitals Dorset NHS Foundation Trust, Poole, GBR; 2 Respiratory Medicine, University Hospitals Dorset NHS Foundation Trust, Poole, GBR; 3 Trauma and Orthopaedics, University Hospitals Dorset NHS Foundation Trust, Poole, GBR; 4 Acute Medicine, University Hospitals of Leicester NHS Trust, Leicester, GBR

**Keywords:** septic arthritis, rare disease or condition, spondylodiscitis, lumbar spine discitis, refusal to walk, pediatric discitis

## Abstract

Discitis in children is uncommon, typically occurring between the ages of two and eight years. The etiology is not established, but it is generally considered a bacterial infection. Symptoms vary with age but commonly include irritability, refusal to walk, and back pain. These various clinical presentations cause delays in diagnosis. We present this case to draw attention and familiarise clinicians with its presentations.

A 17-month-old boy presented with a one-week history of refusal to walk. Examination showed an unusual finding of the child keeping his right leg in a flexed position with hip tenderness. Inflammatory markers were raised. Initially, the patient was managed for suspected septic arthritis of the hip joint. Initial tests were inconclusive, and magnetic resonance imaging (MRI) on day 23 revealed discitis at L4-L5. After a course of antibiotics and monitoring of inflammatory markers, his clinical condition improved.

The mainstay of treatment is antibiotics, with surgery reserved for complicated cases. Discitis has a good prognosis in children.

## Introduction

Discitis is a rare but serious inflammation and infection of the intervertebral discs that affects both adults and pediatric age groups. It affects children aged between two and eight years and typically affects the lumbar spine, though it can affect any part of the spine [1]. The etiology is unknown, but bacterial infection is the common cause [2]. In children, clinical presentation varies with age and often includes non-specific symptoms, which may cause delays and difficulty in diagnosis. Common symptoms in children are irritability, back pain, difficulty walking, and abdominal pain without systemic illness [2].

Magnetic resonance imaging (MRI) is the investigation of choice [1]. Inflammatory markers are often raised and can help in monitoring the response to treatment. The mainstay of treatment is antibiotics for uncomplicated cases, with surgery reserved for complicated cases. Many clinicians are unaware of the disease entity of discitis, which must be included in the differential diagnosis of several acute conditions of childhood. To draw attention to this disorder, we present this report of a 17-month-old boy who presented with a one-week history of refusal to walk. He had been walking independently for the last two months.

## Case presentation

A previously healthy 17-month-old boy presented to the emergency department with a refusal to walk. He had been walking independently for the last two months. On examination, he was lying flat, holding his right hip in a flexed position. There were no obvious swelling, bruising, deformity, wounds, scars, erythema, and rashes. When picked up by his parents from the trolley, his right hip was still in a flexed position. There was tenderness on palpation of the right hip, but no tenderness on palpation of the spine and legs. There was lymphadenopathy in the right inguinal lymph nodes. On passive movement, while attempting to extend the leg at the hip joint, the child began to cry immediately and pulled his leg straight back into flexion. The pain was elicited on internal rotation as well. The examination of the knee and ankle joints was normal. He was afebrile.

**Figure 1:**
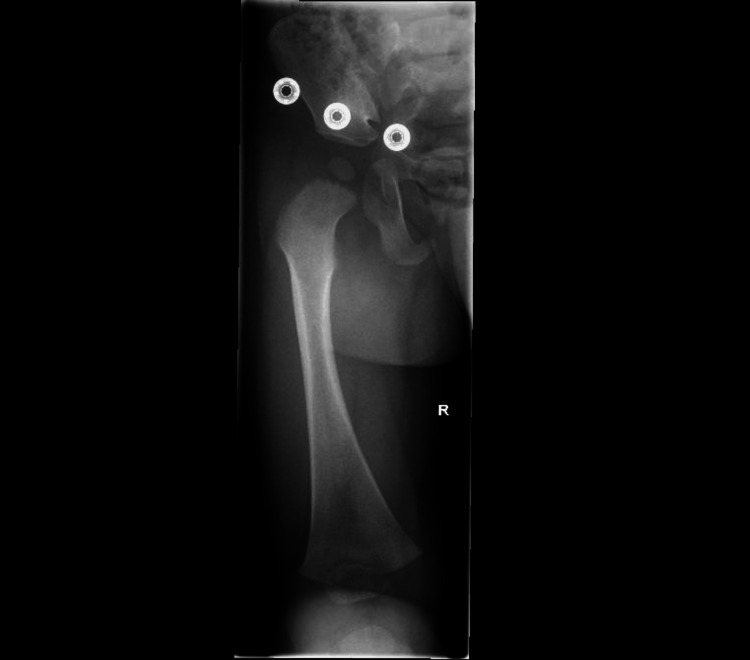
An X-ray of the femur with an anterior-posterior view shows no acute femoral fracture or focal bony lesion. Normal alignment is preserved at the knee joint.

**Figure 2:**
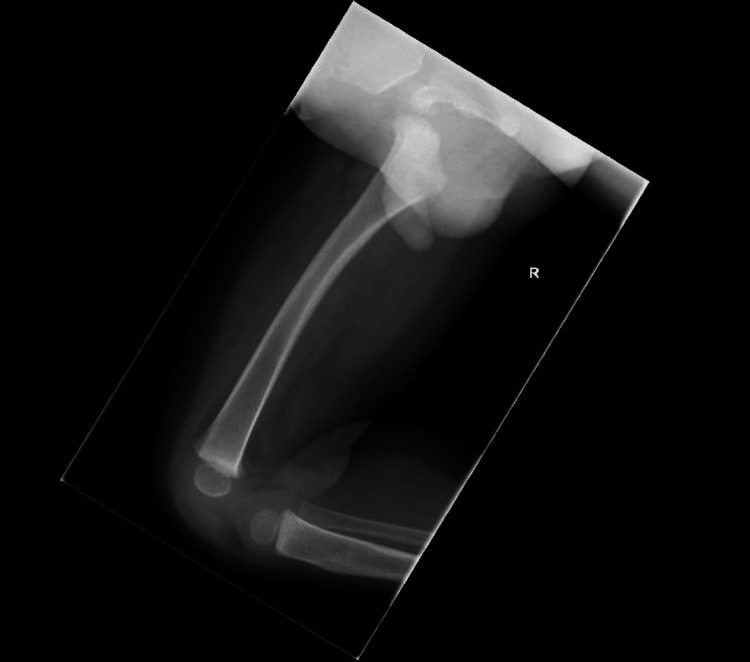
An X-ray of the femur with a lateral view shows no acute femoral fracture or focal bony lesion. Normal alignment is preserved at the knee joint.

**Figure 3:**
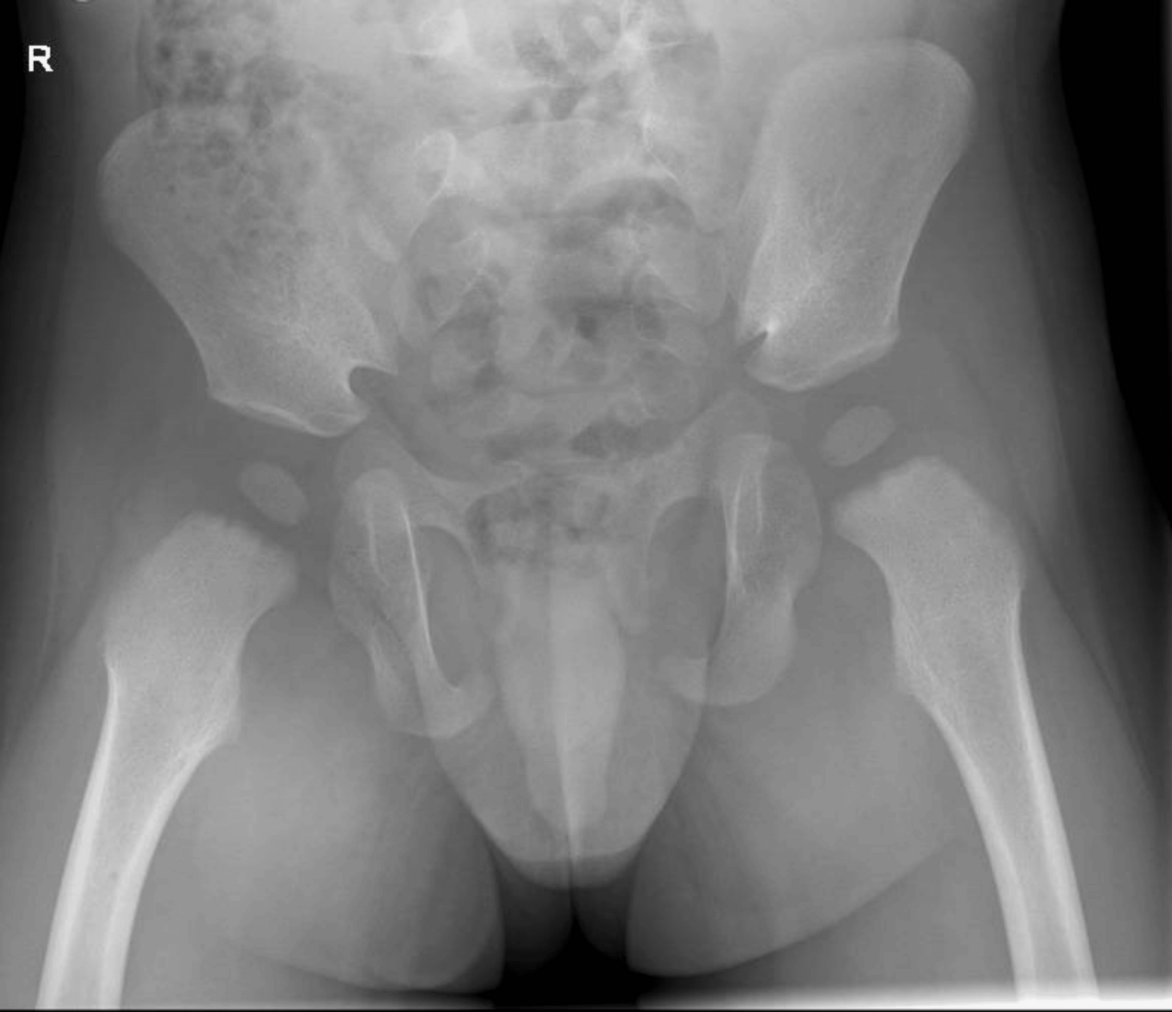
An X-ray of the pelvis shows the femoral capital epiphyses are symmetrical in height and density. Both femoral heads are appropriately covered by their respective normal morphology acetabula. Allowing for minor rotation, there is normal alignment of both hip joints.

An X-ray of the femur (Figure 1 and Figure 2) and pelvis ( Figure 3) was reported as normal. Blood tests showed a white blood cell (WBC) count of 10.6 × 10^9^/L (reference range: 5.0-15.0 × 10^9^/L) and a raised C-reactive protein (CRP) level of 58 mg/L. With differential diagnosis of septic arthritis and transient synovitis of the hip joint, the patient was admitted for an ultrasound scan (USS) of the hip joint and conservative treatment with anti-inflammatory medicines and observations.

The USS of the hip joint reported "Difficult scan due to patient distress and reluctance to move the right hip and knee. No hip joint effusion is identified on either side. There is no large right knee joint effusion". Septic arthritis of the hip joint was ruled out with the help of the USS. The patient was afebrile and not septic. He was discharged with a one-week follow-up for further observation with no clear diagnosis. At the follow-up appointments at one week and two weeks, he was in agonizing pain and reluctant to put his right leg on the floor. He remained afebrile throughout this period.

**Figure 4:**
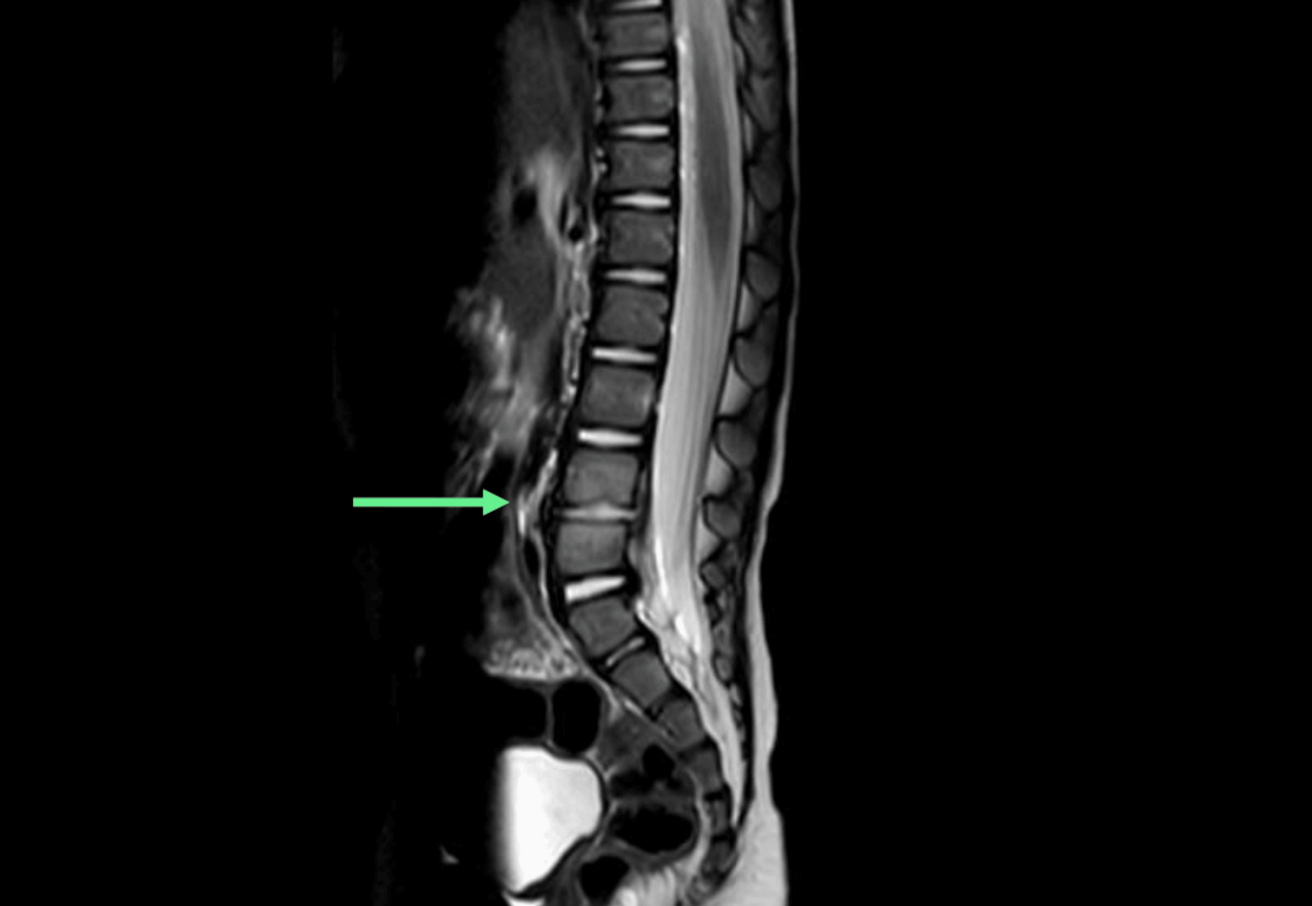
Sagittal view of an MRI of the lumbosacral spine. An arrow points to the disc between the L4 and L5 vertebrae. Fluid signal within the disc with minor loss of disc height and early end plate erosion of L4 and L5 vertebrae. Infective material/pus remains confined within the disc annulus, with no epidural hematoma or canal narrowing seen. MRI: magnetic resonance imaging

An MRI of the spine and hip on day 23 showed discitis at the L4-L5 level with early end plate erosions (Figure 4). On the same day, the WBC count was 16.6 × 10^9^/L (reference range: 5.0-15.0 × 10^9^/L), and the CRP was 50 mg/L. The patient was treated with intravenous ceftriaxone. After two days of antibiotics, the CRP started falling. He was discharged with oral antibiotics for four weeks. At the follow-up after two weeks, he was pain-free and was mobilizing with assistance. The CRP came back to less than 1 mg/L, and the WBC count was 9.8 × 10^9^/L (reference range: 5.0-15.0 × 10^9^/L). 

## Discussion

This report highlights the importance of recognizing discitis to effectively diagnose and manage patients. Familiarity with its unique presentation is necessary to differentiate it from septic arthritis of the hip joint and other conditions with similar presentations.

Discitis is rare in children and is often described in the literature along with spondylodiscitis, which is a simultaneous inflammation of intervertebral discs and adjacent vertebral bodies [3]. It has an estimated prevalence of 1-2 cases per year per 32,500 pediatric hospital evaluations [3]. In another study from a French pediatric orthopedic unit, it was estimated that discitis and spondylodiscitis together accounted for approximately 3% of all admissions related to osteoarticular infections [3].

It most commonly affects children aged between two and eight years [1]. It can affect any part of the spine but most commonly affects the lumbar region in children [2]. The etiology is controversial, but it is generally accepted as a bacterial infection. Blood cultures usually do not show any growth [2]. In our case, all three blood cultures were sterile. Biopsy is not necessary for diagnosis, but when performed, approximately 60% of the patients have positive cultures, with *Staphylococcus aureus* being the most commonly isolated organism. *Kingella kingae* and *Mycobacterium tuberculosis* have also been reported [2]. In most patients, the infection spreads through the hematogenous route [3].

Low incidence, non-specific symptoms, various clinical presentations among different age groups, and children's inability to describe their symptoms make it challenging for clinicians to reach a diagnosis [1]. In infants, it may present with irritability; toddlers may refuse to walk; adolescents often report back pain [4]. Other common features are abdominal pain and vomiting. Usually, patients are afebrile or have a low-grade temperature.

Delays in diagnosis have been reported from two days to six months [3,5]. Initial wrong diagnosis is also not uncommon [5,6]. Similarly, in our case, the patient presented with a refusal to walk, an abnormal position of the right hip, and raised inflammatory markers. The patient was initially treated for septic arthritis, followed by a period of observation until an MRI suggested discitis.

The purpose of this report is to draw attention to this disorder. This will help in reaching the diagnosis early and reducing unnecessary and unwarranted investigations.

In our case, the patient was constantly holding his right hip in a flexed position, which was an unusual examination finding that is difficult to find in the literature. Common examination findings are tenderness on spine palpation, decreased range of motion of the spine, loss of lumbar lordosis, and hip pain and stiffness [2].

Most often, the WBC count is in the high normal range, and CRP varies from normal to slightly raised. Erythrocyte sedimentation rate (ESR) is raised in more than 90% of patients [3,7,8]. In our case, the WBC count was normal, and CRP was raised. We used CRP to monitor the response to treatment, which showed an improving trend after starting treatment. MRI is the investigation of choice for suspected cases of discitis [2]. It helps detect discitis, excludes similar conditions like osteomyelitis, and can provide information regarding complications [8]. Plain radiographs in the early stages of the disease are normal. After one week or more, they may show intervertebral space narrowing [8]. In our case, an X-ray of the pelvis and femur and an USS of the right hip joint for suspected septic arthritis were performed, which did not show any abnormalities. MRI of the spine, femur, and pelvis was performed on day 23, which confirmed the diagnosis of discitis at the L4-L5 level.

There are no standard guidelines for the management of discitis in children. To achieve a better outcome, the literature recommends initial management with intravenous antibiotics until the patient shows clinical improvement, followed by a switch to oral antibiotics as compared to oral antibiotics alone [8]. Empirical antibiotic therapy targeting *Staphylococcus aureus* and a third-generation cephalosporin are recommended. Recommendations for the duration of antibiotics vary, but it should be one to two weeks of parenteral antibiotics followed by four to six weeks of oral antibiotics. Improvement in CRP can be used to guide the duration of treatment [8]. In our patient, we administered intravenous ceftriaxone for four days, which showed significant improvement in CRP, leading to a change of antibiotics to oral co-amoxiclav for four weeks. The patient's symptoms resolved after two weeks, and CRP returned to normal. Rest, immobilization with casting, and analgesia may assist with pain control.

The long-term prognosis is usually good, but anomalies of the disc space and adjacent vertebrae are commonly seen on long-term follow-up.

## Conclusions

Discitis is rare in children. Various clinical presentations and children's inability to verbalize their symptoms make it difficult to diagnose. It can easily mimic septic arthritis of the hip and can divert a clinician's attention away. This report highlights the importance of considering discitis as a differential diagnosis in a child presenting with a refusal to walk. There was an unusual finding observed in the examination, where our patient was constantly holding the right hip in a flexed position, which further needs validation with research work. MRI is very useful in diagnosis, and a 4-6-week course of antibiotics is the mainstay of treatment. To avoid unnecessary delays and improve patient care, we suggest that there is a need for standard guidelines for the investigation and management of this disease, which will be an important step forward.
